# *In vitro* susceptibility of eighteen clinical isolates of human monkeypox virus to tecovirimat

**DOI:** 10.1590/0074-02760230056

**Published:** 2023-07-10

**Authors:** Desiree dos Santos Nunes, Luiza M Higa, Régis Linhares Oliveira, Lendel Correia da Costa, Larissa Maciel Bomfim, Cássia Cristina Alves Gonçalves, Diana Mariani, Dennis E Hruby, Carolina Moreira Voloch, Terezinha Marta Pereira Pinto Castiñeiras, Amilcar Tanuri, Clarissa R Damaso

**Affiliations:** 1 Universidade Federal do Rio de Janeiro, Instituto de Biofísica Carlos Chagas Filho, Rio de Janeiro, RJ, Brasil; 2 Universidade Federal do Rio de Janeiro, Instituto de Biologia, Departamento de Genética, Rio de Janeiro, RJ, Brasil; 3 SIGA Technologies, Inc., Corvallis, OR, USA; 4 Universidade Federal do Rio de Janeiro, Núcleo de Enfrentamento e Estudos de Doenças Infecciosas Emergentes e Reemergentes, Rio de Janeiro, RJ, Brasil

**Keywords:** ST-246, TPOXX, mpox, vaccinia virus, poxvirus

## Abstract

**BACKGROUND:**

In 2022, an outbreak of mpox that started in European countries spread worldwide through human-to-human transmission. Cases have been mostly mild, but severe clinical presentations have been reported. In these cases, tecovirimat has been the drug of choice to treat patients with aggravated disease.

**OBJECTIVES:**

Here we investigated the tecovirimat susceptibility of 18 clinical isolates of monkeypox virus (MPXV) obtained from different regions of Brazil.

**METHODS:**

Different concentrations of tecovirimat were added to cell monolayers infected with each MPXV isolate. After 72 hours, cells were fixed and stained for plaque visualization, counting, and measurement. The ortholog of F13L gene from each MPXV isolate was polymerase chain reaction (PCR)-amplified, sequenced, and the predicted protein sequences were analyzed.

**FINDINGS:**

The eighteen MPXV isolates generated plaques of different sizes. Although all isolates were highly sensitive to the drug, two showed different response curves and IC_50_ values. However, the target protein of tecovirimat, F13 (VP37), was 100% conserved in all MPXV isolates and therefore does not explain the difference in sensitivity.

**MAIN CONCLUSIONS:**

Our results support screening different MPXV isolates for tecovirimat susceptibility as an important tool to better use of the restricted number of tecovirimat doses available in low-income countries to treat patients with mpox.

Monkeypox virus (MPXV) belongs to the genus *Orthopoxvirus*, family *Poxviridae*, and causes a pustular disease recently renamed by the World Health Organization (WHO) as mpox.^1^ Since April 2022, an outbreak of mpox that started in European countries has spread to more than 100 countries worldwide with an exceptional chain of continuous human-to-human transmission.^2^ ^,^ ^3^ As of March 13, 2022, more than 86,200 cases were reported, with 105 deaths. Brazil has more than 10.840 cases and 15 deaths. This is the second highest number of deaths by mpox, matched by Peru and behind only the United States (38 deaths). However, the lethality rates in Brazil and the United States are similar, 0.14% and 0.13%, respectively, while in Peru it reaches 0.4%.^4^ Although the number of new weekly cases has decreased in most WHO regions in recent months, the curves reveal a long outbreak tail in different subregions of the Americas. Also worrying, the number of weekly cases in Central America did not drop sharply during this period, but shows some stability over the last epidemiological weeks. As for Mexico, there was even a recent increase in the number of mpox cases.^5^ In Africa, virus spread to different countries is still observed.^6^ These facts together with the inequity of supply of vaccines and antiviral treatments to developing and low-income countries have supported WHO’s decision to maintain the current mpox outbreak as Public Health Emergency of International Concern.^6^

The clinical presentation of mpox in the 2022 outbreak has varied from a few pustular lesions in the body to lesions, sometimes exuberant, in the perioral, genital, and perianal areas and intense pain. Other severe symptoms are proctitis, rectal bleeding, penis edema, and aggravated ocular infection.^3^ Reinfection has been described,^7^ ^,^ ^8^ and pre-symptomatic and oligosymptomatic individuals may play a role in sustained chains of transmission.^9^ ^,^ ^10^

Currently, few treatments are available for mpox, such as brincidofovir and cidofovir, both inhibitors of the viral DNA polymerase,^11^ and tecovirimat (ST-246), which targets the release of the extracellular virus progeny from infected cells.^12^ ^,^ ^13^ Among the available options, tecovirimat has been the drug of choice in many countries for treating patients with critical conditions or with the potential to become severely ill.^14^ The drug is a synthetic small molecule that was developed by SIGA Technologies as an antiviral against smallpox in the early 2000s.^13^

The target of tecovirimat is VP37 (F13), a 37-kDa palmitoylated phospholipase encoded by the F13L gene (according to the vaccinia virus strain Copenhagen nomenclature) and highly conserved among different orthopoxviruses.^12^ ^,^ ^13^ In infected cells, VP37 is anchored in the membranes of the trans-Golgi network and early endosomes, which wraps part of the intracellular mature virus (MV) progeny to become wrapped viruses (WV), which in turn are addressed to the cytoplasmic membrane for virus release as extracellular viruses (EV).^15^ VP37 is crucial for MV wrapping and the subsequent release of EV from infected cells.^16^ EVs have important roles in both cell-to-cell and long-distance spread of infection. Therefore, F13L knockout vaccinia viruses (VACV) or VACV mutated in important VP37 functional motifs/residues produce tiny plaques due to poor cell-to-cell spread, are highly deficient in spreading infection in cell culture and highly attenuated in mouse infection.^10^ ^,^ ^15^ ^,^ ^16^ Tecovirimat drastically reduces virus spread by impairing VP37-driven virus wrapping.^12^ ^,^ ^17^ ^,^ ^18^ Several mutations in F13L have been identified in tecovirimat-resistant orthopoxviruses and shown to confer resistance to the drug.^12^

Over the years, extensive laboratory data have proved the efficacy of tecovirimat against different orthopoxviruses, including MPXV, in cell culture assays and animal models.^17^ ^,^ ^18^ ^,^ ^19^ In 2018, the FDA approved the use of tecovirimat (TPOXX) to treat smallpox, but its use to treat mpox is authorized under an expanded access program for an investigational drug.^20^ In the EU, tecovirimat received marketing authorization to treat monkeypox in January 2022. However, because of the lack of clinical data on tecovirimat efficacy in infected patients, its use has been authorized under exceptional circumstances.^20^ In Brazil, the use of tecovirimat has been approved on compassionate terms to treat mpox, but so far, the distribution has been limited due to the restricted number of treatments available. To fill in the missing data on the use of tecovirimat to treat infected patients, numerous clinical trials are underway around the world to assess its safety and efficacy against human mpox.^21^ ^,^ ^22^

The clinical success of tecovirimat treatment is undoubtedly multifactorial, but a minimum requirement is that MPXV isolates should be susceptible to the antiviral effect of the drug. This fact underscores the importance of investigating the sensitivity of different MPXV clinical isolates to tecovirimat. In this context, MPXV currently circulating in Brazil is probably genetically diverse as there have been multiple virus entries into the country from different regions of Europe and North America since the beginning of the outbreak.^23^ Therefore, here we investigated the *in vitro* efficacy of tecovirimat in inhibiting the replication of 18 clinical isolates of MPXV obtained from patients from different regions of Brazil from June to August 2022.

## MATERIALS AND METHODS

*Virus detection and diagnostics* - The Federal University of Rio de Janeiro (UFRJ), represented by the Working Group on Monkeypox Preparedness [Núcleo de Enfrentamento e Estudos de Doenças Infecciosas Emergentes e Reemergentes (NEEDIER), the Laboratory of Molecular Biology of Virus, and the Laboratory of Molecular Virology], has been designated by the Brazilian Ministry of Health as one of the reference centers for molecular diagnostics of mpox and MPXV isolation in cell culture for five states of Brazil [Rio de Janeiro (RJ), Mato Grosso (MT), Mato Grosso do Sul (MS), Goiás (GO), and Espírito Santo (ES)] and the Federal District (Brasília, capital of Brazil) until October, 2022. Samples from patients with suspected mpox were sent to UFRJ by Central Public Health Laboratories located in each state. Dry swabs collected from wet lesions or skin lesion fragments were incubated with phosphate-buffered saline (PBS), an aliquot was stored at -80ºC for virus isolation and the remaining sample was processed for DNA extraction using a KingFisher Flex System^®^ (Thermo Fisher) and the MagMax Viral/Pathogen Nucleic Acid Isolation Kit (Thermo Fisher), according to the manufacturer’s protocol. MPXV DNA was detected by a triplex polymerase chain reaction (PCR) assay (Exxtend) aimed at the detection of any orthopoxvirus,^24^ clade I and II MPXV,^25^ and the human RNase P gene as endogenous control, except for the first positive sample (MPXV008) that was detected by the orthopoxvirus primers and probe only,^24^ followed by conventional PCR amplification^26^ and nanopore sequencing (Oxford Nanopore Technologies) of the topoisomerase gene. Positive samples had cycle threshold (Ct) values ≤ 37.

*Virus isolation in cell culture and stock titration* - All procedures involving experimental manipulation and propagation of infectious MPXV were performed in a biosafety level (BSL)-3 containment laboratory. In all experiments, we used monkey kidney BSC-40 cells (kindly provided by Richard Condit, University of Florida, USA) grown in monolayers under high-glucose DMEM with 7% fetal bovine serum (FBS) (Thermo Fisher) at 37ºC/5%CO_2_. For virus isolation, 50-100 µL of the PBS-eluted material from the swabs were incubated with BSC-40 monolayers until full cytopathic effect was achieved, usually within 48 to 72 hours.^27^ Cells were harvested and resuspended in PBS for further storage at -80ºC. Virus stocks were titrated by plaque assay and titers are expressed as the average number of plaque-form units per mL (PFU/mL).^17^ ^,^ ^27^

*Effect of tecovirimat on virus plaque formation* - A 10 mM tecovirimat stock solution (SIGA Technologies) was prepared in 100% dimethyl sulfoxide (DMSO) and stored at -20ºC. Working solutions (1,000 x concentrated) were freshly prepared by diluting the stock solution in 100% DMSO before each experiment.^17^ Subconfluent BSC-40 cells grown in 6-well plates (1 x 10^6^ cells/well) were infected with 300 PFU of each clinical isolate. After adsorption, cells received Dulbecco’s Modified Eagle Medium (DMEM) with 7% FBS containing 0.1% DMSO (vehicle control) or 2, 4, 5, 7.5, and 10 nM tecovirimat in duplicate. After 72 hours, cells were fixed and stained for plaque visualization.^17^ The experiments were repeated three times independently.

Plates were photographed and images were equally pretreated to standardize position and rotation using ImageJ software (http://imagej.nih.gov/ij). Individual well images were cropped and trimmed to square images using the Viridot Image Formatting Program.^28^ Viral plaques were counted and measured by setting the following parameters: light setting = saturation; blur image =2; remove strings/fibers in image = 0.1; cut well edges = 72; apply contrast to image based on background and plaque intensity = 1.4 and 4; select difference in pixel value to distinguish plaque from background = 0.08; size (in pixels) of the window for applying the thresholding algorithm to image = 10; dilate plaques to ensure they are counted as single objects = 5; cut overlapping plaques, so they are counted separately = 1; define the minimum and maximum pixel size to count as a plaque = 0 and 1436, respectively. The size of viral plaques in pixels was converted to mm^2^ by calculating a conversion factor for each well image, as follows: 960 (well area in mm^2^) /well are in pixels = conversion factor multiplied by each viral plaque area in pixels for that particular image. At least 550 random plaques were measured for each assay point, as specified in the Figure legend.

*DNA extraction and nanopore sequencing* - Total DNA was extracted from 50 µL of each clinical isolate virus stock using the QIAamp DNA Mini Kit (Qiagen) according to the manufacturer’s instructions. The F13L gene was PCR-amplified using the primer set, reaction and cycling conditions described elsewhere.^17^ PCR products were purified and quantified using fluorometry with the Qubit dsDNA High Sensitivity Assay (Life Technologies). The library was prepared using Rapid barcoding kit 96 (SQK-RBK110.96), following the manufacturer’s protocol, and then loaded onto an FLO-MIN106 flow cell on a MinION device (Oxford Nanopore Technologies). FASTQ files were demultiplexed, trimmed, and exported by MinKNOW API standard setup. The barcoded FASTQ files were aligned and mapped to a reference genome (GenBank accession no. NC_063383.1) using the Geneious mapper. Geneious Prime 2022.2 was also used for computing the number of mapped reads and mean cover­age depth, and consensus sequence generation.

The datasets generated and/or analyzed during the current study are available in the GenBank repository, BioProject ID PRJNA925815 (http://www.ncbi.nlm.nih.gov/bioproject/925815), BioSample accessions: SAMN32810014, SAMN32810015, SAMN32810031, SAMN32810016, SAMN32810017, SAMN32810018, SAMN32810019, SAMN32810020, SAMN32810021, SAMN32810022, SAMN32810023, SAMN32810024, SAMN32810025, SAMN32810026, SAMN32810027, SAMN32810029, and SAMN32810030. Complete sequences of the F13L gene obtained for the 18 clinical isolates received the following GenBank accession numbers: OQ197864 (MPX 008); OQ197865 (MPX 034); OQ197882 (MPX 041); OQ197866 (MPX 062); OQ197867 (MPX 068); OQ197868 (MPX 069); OQ197869 (MPX 073); OQ197870 (MPX 086); OQ197871 (MPX 088); OQ197872 (MPX 118); OQ197873 (MPX 119); OQ197874 (MPX 120); OQ197875 (MPX220); OQ197876 (MPX 221); OQ197877 (MPX 449); OQ197878 (MPX 450); OQ197879 (MPX 461); OQ197880 (MPX 902).

*F13 alignment and base-by-base analysis* - The predicted amino acid sequences of the F13 orthologs of different MPXV isolates and other orthopoxviruses were aligned using the Mafft server with default parameters (https://mafft.cbrc.jp/alignment/server/). The multi-alignment was submitted to Base-by-base analysis and screened for the presence of SNPs, opting for MPXV isolate 0008 as the reference sequence.^29^ Preserved residues appear in white, and each SNP is represented by a blue bar. The location of important sites and motifs was done using Motif (https://www.genome.jp/tools/motif/), associated with manual inspection based on previous work.^17^ ^,^ ^30^ The Genbank accession numbers used in this work are as follows: MPXV_Brazil_1 (ON751962); MPXV_Brazil_2 (ON880413); MPXV_UK_2022 (OP415195); MPXV_France_2022 (MT903341); MPXV_UK_2018 (MT903344); MPXV_USA_039_2003 (DQ011157); MPXV-LBR_1970 (DQ011156); vaccinia virus (VACV)_WR (AY243312) and VACV_IOC_B141 (KT184690); variola virus (VARV)-BGD74 (DQ441422) and VARV-Gar-1966 (DQ441419); and camelpox (CMLV)_CMS (AY009089).

*Ethical considerations* - This study was approved by the Research Ethics Committee of the Clementino Fraga Filho University Hospital of the Federal University of Rio de Janeiro, under the protocol number CAAE 62281722.5.0000.5257.

*Statistical analyses* - All plots and statistical analyses were performed using GraphPad Prism^®^ vs 8.02 (GraphPad Software). To analyze the inhibition of plaque number and size, we used the two-way ANOVA with Tukey’s and Sidak’s multiple comparisons tests. We used the unpaired Student’s test to compare IC_50_ values for MPXV isolates 221 and 450. The slope of the curves for plaque size reduction was analyzed by linear regression.

## RESULTS

**Fig. 1:**
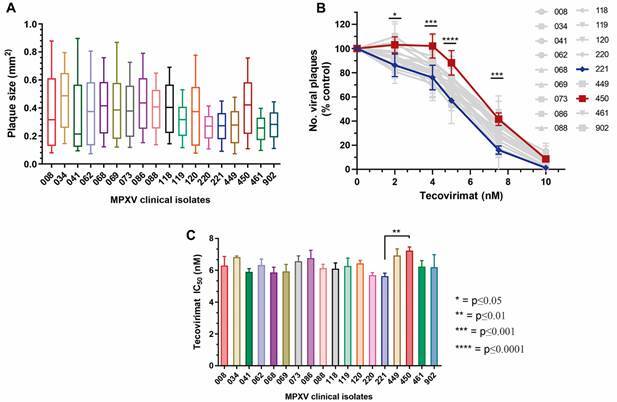
diversity of monkeypox virus (MPXV) plaque phenotype and effect of tecovirimat on virus replication: BSC-40 cells were infected with 300 plaque-forming unit (PFU) of each MPXV clinical isolate for 72 hours when cells were fixed and stained. (A) Box and Whiskers (10-90 percentile) plot showing the size diversity of nearly 1,500 viral plaques (minimum of 550 plaques) measured for each isolate in three independent experiments. Median ± standard deviation (SD) is shown. (B, C) Infection proceeded in the presence of different concentrations of tecovirimat for 72 hours when cells were fixed and stained. Data are represented as mean ± SD. (C) Half maximal inhibitory concentration (IC_50_) was calculated based on the linear regression equation formula for each dose-response curve. All experiments were performed in triplicate.

The Federal University of Rio de Janeiro is one of the reference centers designated by the Brazilian Ministry of Health to conduct the molecular diagnosis of mpox and virus isolation from clinical material. Until October 2022, the University received clinical material from five states and the Federal District. For virus isolation in cell culture, we selected 18 PCR-positive samples with Ct < 30, representing three clinical isolates from each of the six locations (Table). Infection of BSC-40 cells with 300 PFU of each cultured MPXV isolate revealed different plaque sizes that did not directly correlate with sample origin or collection date (Fig. 1A). The phenotype of orthopoxvirus plaques is a genetic-driven feature related to the expression of numerous viral genes.^15^ Therefore, differences in plaque size were taken as indicative of genetic variability between the MPXV isolates.

**TABLE:** Clinical isolates of monkeypox virus used in this study

Clinical isolate	Date of collection	Ct values for swab samples^*a*^	Yield^*b*^ (PFU/mL)	Origin^*c*^ (State of Brazil)	Sex	Year of birth
008	6/12/2022	27.0	8.45 ˟10^8^	RJ	Male	1982
0034	6/28/2022	19.0	1.00 ˟10^9^	DF	Male	1991
0041	7/2/2022	11.33	1.07 ˟10^9^	RJ	Male	1991
0062	7/5/2022	13.7	1.00 ˟10^9^	RJ	Male	1989
0068	7/4/2022	10.69	1.70 ˟10^9^	GO	Male	1988
0069	7/4/2022	11.28	1.30 ˟10^9^	GO	Male	1988
0073	7/6/2022	11.32	1.20 ˟10^9^	DF	Male	1988
0086	7/5/2022	11.8	1.20 ˟10^9^	DF	Male	1994
0088	7/6/2022	18.25	1.30 ˟10^9^	GO	Male	1980
0118	7/6/2022	18.2	1.40 ˟10^9^	MS	Male	1981
0119	7/6/2022	18.46	1.30 ˟10^9^	ES	Male	1990
0120	7/7/2022	24.4	4.80 ˟10^8^	ES	Male	1977
0220	7/18/2022	18.13	1.80 ˟10^9^	MS	Male	1986
0221	7/14/2022	17.17	1.60 ˟10^9^	MS	Male	1994
0449	8/1/2022	17.32	1.10 ˟10^9^	MT	Male	1988
0450	8/1/2022	19.47	1.84 ˟10^9^	MT	Male	1994
0461	7/28/2022	14.87	9.65 ˟10^8^	ES	Male	1992
0902	8/2/2022	15.85	1.40 ˟10^9^	MT	Male	1994

*a*: cycle threshold (Ct) values correspond to results obtained for DNA isolated from original swab samples. Diagnostics were done by using generic primers for monkeypox virus (MPXV), except for clinical isolate # 008, which was detected by using primers for *Orthopoxvirus* followed by DNA sequencing that confirmed the presence of MPXV genome; *b*: yield obtained after the first passage of virus isolation in BSC-40 cells, except for isolates 008 and 450 that a second passage in cell culture was necessary to obtain a generalized cytopathic effect in the monolayers; *c*: states of Brazil: RJ, Rio de Janeiro; GO, Goiás; MT, Mato Grosso; MS, Mato Grosso do Sul; ES, Espírito Santo. DF (Federal District) refers to the Federal Capital of Brazil, Brasília.

Next, we investigated the sensitivity of MPXV isolates to non-cytotoxic concentrations of tecovirimat^17^ (Fig. 1B). In general, most MPXV isolates exhibited very similar response curves that led to dose-dependent inhibition of viral plaque numbers at nanomolar concentrations and > 85% inhibition at 10 nM. However, isolates 221 and 450 responded differently to tecovirimat in the range of 2 to 7.5 nM (Fig. 1B). The difference was statistically significant and resulted in slightly but significantly different IC_50_ (50% inhibitory concentration) values of 5.6 ± 0.32 nM and 7.2 ± 0.40 nM for isolates 221 and 450, respectively (Fig. 1C). Pre-treatment of cells for 2 h with 10 nM tecovirimat did not inhibit virus plaque formation and did alter the sensitivity of MPXV isolates 221 and 450 to 5 nM tecovirimat added after infection (data not shown). For all 18 isolates, we observed no statistically supported correlation between plaque size and IC_50_ (Pearson’s correlation coefficient r = 0.2975, p = 0.2305). At 100 nM, > 99.4% inhibition of the production of infectious progeny was equally observed for both 221 and 450 isolates (data not shown).

**Fig. 2:**
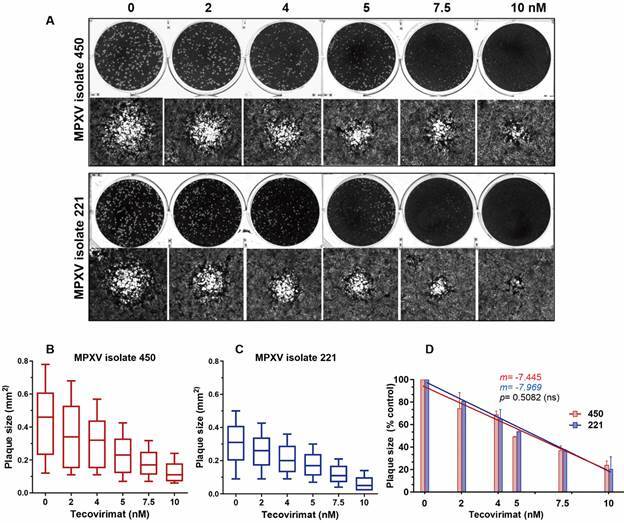
effect of tecovirimat on plaque size of monkeypox virus (MPXV) isolates 450 and 221: BSC-40 cells were infected with 300 plaque-forming unit (PFU) of each of the 18 clinical isolates of MPXV for 72 hours in the presence of different concentrations of tecovirimat when cells were fixed, stained, and photographed. (A) Representative images of wells with viral plaques generated in the cell monolayers (upper row) and size-representative plaques photographed at 4x magnification. (B, C) Box and Whiskers (10-90 percentile) plot showing the size diversity of at least 590 plaques (maximum of 830 plaques) measured for each concentration of tecovirimat in three independent assays. Median ± standard deviation (SD) is shown. (D) Percent of control plot values obtained in B and C expressed as mean ± SD. *m* indicates the slope values for each linear regression line and the p-value indicates that the difference between the slopes is not significant (ns). All experiments were performed in triplicate.

Tecovirimat also reduced the size of viral plaques in all 18 MPXV isolates. However, we focused our analysis on isolates 450 and 221, as shown in Fig. 2A. MPXV 450 generated viral plaques on average 1.54 times larger than MPXV 221 (p < 0.0001), and, for both isolates, plaque sizes progressively decreased with increasing concentrations of tecovirimat (Fig. 2A, C). However, no statistically significant differences were observed between the slopes of the reduction curves (Fig. 2D). Furthermore, the percentage reduction for each concentration of tecovirimat, compared to its respective mock control, was similar for both isolates (Fig. 2D). At 10 nM, plaque sizes were 24.09 ± 2.58 % and 15.26 ± 6.17 % of their original sizes for MPXV isolates 450 and 221, respectively, and the difference between the isolates was not statistically significant (Fig. 2D).

**Fig. 3:**
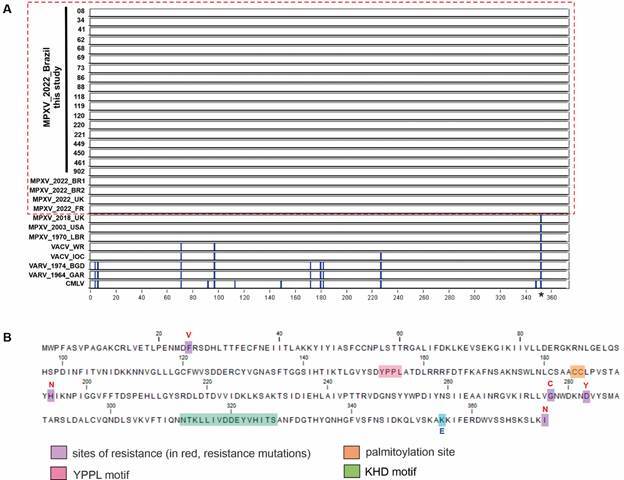
multi-alignment of F13 amino acid sequences: (A) The multi-alignment was screened for single nucleotide polymorphisms (SNPs shown in blue) using Base-by-base, opting for monkeypox virus (MPXV) isolate 08 as the reference sequence. Preserved residues are shown in white. The red dotted box indicates sequences from the 2022 mpox outbreak. Asterisk (and blue residue in B) indicates the lysine that replaced glutamic acid in all 2022 sequences. (B) Representative F13 protein sequence from Brazilian MPXV isolates, highlighting key motifs critical to VP37 functionality and known tecovirimat resistance residues.

Next, we amplified and sequenced the F13L gene from the 18 MPXV isolates. Analysis of predicted amino acid sequences of VP37 revealed 100% sequence conservation among all 18 Brazilian MPXV isolates (Fig. 3A), including the preservation of all residues and motifs critical to VP37 functionality (Fig. 3B). The sequences lack mutations already described to confer resistance to tecovirimat in other orthopoxviruses^12^ (Fig. 3B), but they have the E353K substitution (Fig. 3A-B, asterisk) detected in all MPXV sequences from the 2022 multinational outbreak.^31^ ^,^ ^32^ ^,^ ^33^

## DISCUSSION

In this work we have investigated the *in vitro* sensitivity of 18 cultured isolates of MPXV from different regions of Brazil. All isolates were highly sensitive to tecovirimat with similar dose-response curves, except for two isolates that revealed significant differences in the IC_50_ values and in plaque size reduction induced by tecovirimat treatment. Differences in tecovirimat sensitivity in MPXV isolates with identical VP37 sequences are not entirely unexpected. Previous reports have shown that different strains or clinical isolates of orthopoxviruses, such as vaccinia and variola viruses, can have identical VP37 sequences and show different tecovirimat response curves.^17^ ^,^ ^18^ So far, VP37 is the only viral protein with a recognized role in the mechanism of action of tecovirimat.^12^ However, other factors may be affecting virus sensitivity, such as the level of VP37 expression during the virus cycle and the production of extracellular viruses by each orthopoxvirus strain.^34^ In the case of the 18 MPXV isolates studied in this work, all have identical promoter sequences, suggesting similar F13L transcription rates (data not shown).

The size of viral plaques is affected by the expression of numerous genes, including F13L.^15^ ^,^ ^35^ As the F13L sequences and promoter regions of the 18 isolates were identical, alterations in other single or multiple genes may play a role in the plaque size difference observed for the MPXV isolates. Further sequencing of the complete genomes is necessary to unveil the genetic diversity of the isolates, associated with in-depth analyses of SNPs and INDELs with marker rescue experiments to determine the gene involved in the small plaque phenotype of isolate 221. The size of viral plaques also reflects viruses’ ability to spread in cell culture; viruses with a small plaque phenotype spread poorly in cell culture.^15^ Therefore, viruses we larger plaques would be expected to be less sensitive to tecovirimat as the drug targets VP37, which is involved in virus release from cells. However, no statistically supported correlation was observed between the size of viral plaques generated by all 18 isolates and their IC_50_ values.

The eighteen VP37 sequences analyzed in this work have the E353K mutation that is found in all MPXV genomes of the 2022 outbreak, including MPXV isolates from Germany^31^ and France.^32^ This substitution does not appear to play a role in tecovirimat sensitivity because the 18 isolates from Brazil, as well as the MPXV isolates from France and Germany, are highly susceptible to tecovirimat at nanomolar concentrations.^31^ ^,^ ^32^ MPXV isolates from previous years have K (lysine) instead of E (glutamic acid) in position 353.^33^

Our study has some limitations. *In vitro* analysis of an antiviral always limits the study conclusions. Therefore, testing tecovirimat susceptibility in well-tuned animal models such as non-human primates, CAST/EiJ mice or praire dogs^36^ infected with these MPXV isolates, would add robustness to our work. However, MPXV is a BSL-3 pathogen, which limits animal experimentation to BSL-3 animal facilities. Likewise, investigating susceptibility to tecovirimat in patients as part of clinical trials will also be extremely important, and several protocols are being implemented in different countries to address this issue.^21^ ^,^ ^37^ ^,^ ^38^ The number of clinical isolates is also a limitation of our study. However, the isolation and manipulation of MPXV, a BSL-3 pathogen, requires strict biosafety protocols that restrict the workload. Despite this, to the best of our knowledge, our study has investigated, to date, the largest number (18) of cultured clinical isolates of MPXV from the 2022 outbreak for *in vitro* susceptibility to tecovirimat.^31^ ^,^ ^32^ ^,^ ^39^ However, it is important to note that our results apply to MPXV isolated in Brazil and may not reflect the drug sensitivity of MPXV isolates from other countries, although isolates from Germany, Canada and France were also highly susceptible to tecovirimat.^31^ ^,^ ^32^ ^,^ ^39^

In summary, our work shows that the 18 MPXV isolates are highly sensitive to tecovirimat at the nanomolar scale, and the observed differences between some isolates, particularly MPXV 221 and MPXV 450, should be further investigated to assess whether it has any biological relevance in the context of clinical use of tecovirimat. In this case, additional drug sensitivity testing should probably be incorporated into practice, in addition to F13L sequence analysis, in order to infer the susceptibility of an MPXV isolate to tecovirimat. It is also important that other isolates, especially those isolated from patients not responding to tecovirimat treatment, be analyzed in more detail.

Our results support screening different MPXV isolates for tecovirimat susceptibility as an important tool that may contribute to better use of the restricted number of tecovirimat doses available in low-income countries to treat patients with mpox. The scenario of long outbreak tails observed in several countries, including in South America, is exacerbated by the difficulty of breaking person-to-person transmission of the virus due to the limited availability of orthopoxvirus vaccines worldwide and the silent transmission by presymptomatic and oligosymptomatic patients.^2^ ^,^ ^9^ ^,^ ^10^
